# A novel echocardiography method to assess upper body systemic blood flow in preterm infants and comparison with superior vena cava flow measurement

**DOI:** 10.1007/s00431-023-04866-9

**Published:** 2023-02-16

**Authors:** Federico Schena, Rossella Iannotta, Vito D’Andrea, Gaia Francescato, Alessandra Mayer, Giuseppina Mancini, Giorgia Prontera, Fabio Mosca, Gianni Vento

**Affiliations:** 1 Neonatal Intensive Care Unit-AO SS, Antonio e Biagio e C. Arrigo Hospital, Alessandria, Italy; 2 Department of Woman and Child Health and Public Health, Catholic University of Sacred Heart, Fondazione Policlinico Universitario “A. Gemelli” IRCCS, Rome, Italy; 3 NICU, Fondazione IRCCS Ca’ Granda Ospedale Maggiore Policlinico, Milan, Italy; 4 Department of Clinical Sciences and Community Health, University of Milan, Milan, Italy

**Keywords:** Neonatal hemodynamic, Superior vena cava flow, Preterm infants

## Abstract

To evaluate feasibility, reproducibility, and prognostic value of a new echocardiographic method to assess systemic arterial blood flow directed to the upper part of the body (UBAF, upper body arterial flow) alternative to superior vena cava flow (SVCF) measurement. We performed echocardiographic evaluations in 106 infants in the first 2 days of life to obtain SVCF, left ventricle output (LVO), UBAF, and standard parameters of patent ductus arteriosus (PDA) significance. UBAF was calculated by subtracting from LVO the aortic arch blood flow measured immediately distally to the origin of the left subclavian artery. Main outcome measures: UBAF and SVCF agreement was assessed by Bland–Altman analysis in terms of bias, limits of agreement and repeatability index. The Intraclass Correlation Coefficient was used to measure the strength of inter-rater agreement. The agreement between UBAF and SVCF was high. The Concordance Correlation Coefficient (CCC) was 0.7434. (CCC 0.7434, 95% C.I. [0.656, 0.8111]). There was a good absolute agreement between the two raters ICC = 0.747; *p* value < 0.0001; 95%CI [0.601; 0.845]. Adjusting for confounding factors (BW, GA, PDA) included in the model, there was a statistically significant relationship between UBAF and SVCF.

*Conclusion*: UBAF showed a strong agreement with the SCVF with a better reproducibility. Our data support UBAF as a potentially useful marker of cerebral perfusion in the evaluation of preterm infants.

**Table:** 

**What is Known:** *• Low SVC (superior vena cava) flow in the neonatal period has been associated with periventricular haemorrhage and unfavourable long-term neurodevelopmental outcome.* *• Ultrasound measurement of flow in SVC shows relatively high inter-operator variability.*	
**What is New:** *• Our study highlights how much overlap there is between upper-body arterial flow (UBAF) measurement and SCV flow measurement*. *UBAF is easier to perform and has a strong correlation with better reproducibility.* *• UBAF may replace measurement of cava flow as a method for haemodynamic monitoring of unstable preterm and asphyxiated infants.*

**What is Known:**

*• Low SVC (superior vena cava) flow in the neonatal period has been associated with periventricular haemorrhage and unfavourable long-term neurodevelopmental outcome.*

*• Ultrasound measurement of flow in SVC shows relatively high inter-operator variability.*

**What is New:**

*• Our study highlights how much overlap there is between upper-body arterial flow (UBAF) measurement and SCV flow measurement*. *UBAF is easier to perform and has a strong correlation with better reproducibility.*

*• UBAF may replace measurement of cava flow as a method for haemodynamic monitoring of unstable preterm and asphyxiated infants.*

## Introduction

Approximately 30% of very low birth weight (VLBW) infants develop low systemic blood flow (SBF) in the first few days after birth [1, 2]. A condition of systemic hypoperfusion increases the risk of acute renal failure, intestinal perforation, intraventricular haemorrhage, and unfavourable neurological outcomes in these patients [3]. Therefore, it is mandatory to identify parameters that reflect systemic perfusion and blood flow to districts at risk of ischemic injury [4].

The usual echocardiographic measures of SBF are unreliable in the first days of life because of the persisting shunts (ductus arteriosus and foramen ovale) between systemic and pulmonary circulations, so ventricular outputs do not represent true systemic perfusion [5]. To overcome this limitation, in 2000 Kluckow and Evans proposed the use of superior vena cava flow (SVCF) as a measure of systemic and cerebral perfusion since it represents the venous return of the upper half of the body unaffected by intracardiac or extra-cardiac shunts [6].

We hypothesized that the SVCF measurement could be replaced by a measurement of arterial flow directed to the upper part of the body (upper body arterial flow, UBAF), obtained by the difference between left ventricle output (LVO) and flow in descending aorta, measured immediately distal to the emergence of the left subclavian artery, but proximal to the emergence of the ductus arteriosus (DA), and we hypothesized that this measure might be easier to perform, reproducible, and maintain a similar diagnostic and prognostic value compared to SVC flow measurement. Potential advantages of the UBAF measurement include the minimal changes in flow with breathing, the fact that it can be obtained through a single echocardiographic projection (suprasternal view), the minimal changes in vessel calibre (arterial vessel that does not collapse) and the ability to obtain the measurement even in the case of anatomical variants, abdominal distension or surgical medications. The purpose of the study was to assess the agreement between UBAF and SVCF.

## Methods

This was a 2-center, prospective observational prospective study performed at the Neonatal Intensive Care Unit (NICU) of the IRCCS Foundation Cà Granda Ospedale Maggiore Policlinico of Milan and at the NICU of the IRCCS Foundation Agostino Gemelli Polyclinic of Rome.

All term and preterm infants admitted to the NICU were enrolled in a 12-month period. The echocardiographic examination was performed in the first two days of life. Exclusion criteria were major congenital abnormalities, chromosomal anomalies, and congenital heart defects (CHD) except of patent foramen ovale (PFO) and patent ductus arteriosus (PDA).

Written informed consent was obtained from the parents before each study. The research methods conformed to the standards set by the Helsinki Declaration.

Echocardiographic measurements were performed by two investigators (FS, RI) using Aloka Prosound α7 and Logiq S8 (GE Healthcare) ultrasound machines with 8 and 10 MHz sector probes. The 10 MHz probe was used in infants with birth weight less than 2000 g. The presence of CHD was excluded in every echocardiography.

**Fig. 1:**
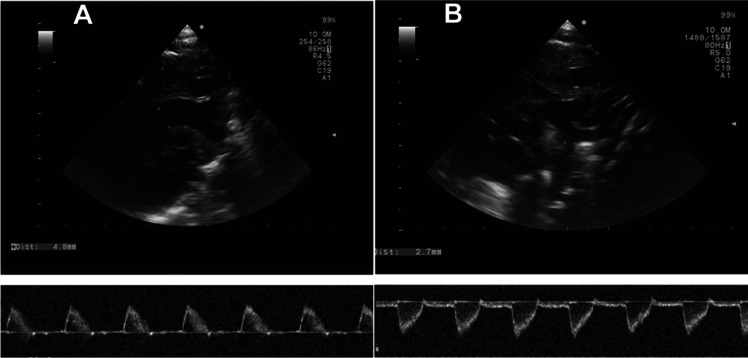
Flows in ascending (**A**) and descending (**B**) aorta measured by US

Measurement of UBAF was obtained from the difference between the flow in the ascending aorta (LVO) and the flow in the pre-ductal descending aorta. Both of these measurements were obtained through the suprasternal view: the ascending aorta, arch, and descending aorta were visualized in a single ultrasound view. The measurement of blood flow velocity in the ascending aorta was obtained by pulse wave doppler, positioning the sample volume in the first portion of the ascending aorta visible through the jugular projection and aligning the ultrasound beam to the flow direction. The measurement was performed on the first portion of the ascending aorta visible through the jugular view. The blood flow velocity was recorded as a function of time and the VTI was calculated (Fig. 1A). The diameter of the ascending aorta was measured from the same view at the point where the perpendicular to the walls of the ascending aorta crosses the right branch of the pulmonary artery. Blood flow velocity in the descending aorta was measured by positioning the sample volume immediately after the emergence of the left subclavian artery and aligning the ultrasound beam to the flow direction. The diameter of the descending aorta was measured in that point and before the possible emergence of the DA (Fig. 1B). When measuring the velocity, care must be taken to ensure that the angle of insonation as parallel as possible to the direction of flow. If the ultrasound beam intersects the blood flow at an angle, the velocity will be underestimated. In clinical practise, an angle of less than 20 is generally considered acceptable as the effect on velocity is negligible. An angle greater than 20 requires an angle correction, which often produces inconsistent results. When assessing flow through the aortic isthmus, the suprasternal or right subclavicular windows usually provide an optimal angle for Doppler interrogation.

SVC diameter was assessed from a modified parasternal long axis view as described by Kluckow and Evans [6]. High-definition zoom was used to focus on the SVC as it begins to open up into the right atrium, with maximum and minimum diameters through the cardiac cycle taken from B-mode images. In all cases, 3–5 consecutive cycles were analysed. To assess flow velocity, a low subcostal view was used, with the ultrasound probe moved caudally until a clear length of the SVC could be seen entering the right atrium, where the pulsed wave Doppler gate was placed, 8–10 cycles were analysed to reduce the impact of respiratory variability.

The following measures were performed during each evaluation: PDA (diameter, flow direction, pulsed doppler pattern, hemodynamic significance); SVCF; UBAF, obtained from the difference between the flow in the ascending aorta (LVO) and flow in the pre-ductal descending aorta. Ultrasound image quality (good or poor) was defined by the operator performing the examination before measurements were taken. A PDA was deemed hemodynamically significant when diameter was > 1.5 mm, the flow pattern was left-to-right pulsatile or growing and the left atrium:aorta ratio was > 1.5 [7].

In a second step, inter-operator agreement in the acquisition of the two different measurements were assessed. Regarding inter-operator agreement, the echocardiographic examination was performed on the same infant by two different operators, and the images were acquired independently by the two operators at 10 min apart. All measurements and flow calculations were performed on images recorded 1 week after the examination. The scan-rescan inter-operator was performed in a sub-cohort of patients (54 infants).

Antenatal and demographic factors were collected at the time of enrolment and the data are reported as means (± SD).

The entire population was studied to evaluate the agreement between UBAF and SVCF. In addition, the following outcomes were studied:

- The Intraclass Correlation Coefficient (ICC) was used to measure the strength of inter-rater agreement.
- the relationship between SCVF and UBAF adjusted for several possible confounding variables such as Ga, BW, and PDA.

### Statistical analysis

Bland–Altman (B-A) analysis was used to calculate and visualize the agreement between the standard SCVF and UBAF measurement. The agreement limits are demonstrated as a 95% confidence interval (95% CI = mean ± 1.96 standard deviations), where the ideal agreement difference between measurements is zero.

The relationship between the two methods (UBAF and SCVF) was further investigated by a multiple regression model adjusted for demographic and clinical variables. The intraclass correlation coefficient (ICC) was used to measure the strength of inter-rater agreement. Then, the intraclass correlation coefficient was computed to assess the agreement between two raters in rating the UBAF measures.

To assess the reproducibility of the new method, the correlation coefficient between the UBAF measured by the two different operators was determined. The correlation coefficient for the standard SCVF method was calculated to compare UBAF and SVCF in terms of reproducibility. The statistical analysis was carried out using MedCalc, GraphPad, SPSS and R version 4.2.2 (R cran 2022).

A sample of 106 subjects achieves 80% power to detect agreement when the confidence level of the LoA is 0.95 and the confidence level of the confidence intervals about the LoAs is 0.95. The maximum allowable difference is 2.5. The mean and standard deviation of the sample differences are anticipated to be 0 and 1.

The study was approved by the Institutional Ethics Committee Ethics Committee of the Fondazione Policlinico Universitario A. Gemelli IRCCS, Rome, Italy (N. 50294/19 ID2898.)

## Results

**Fig. 2:**
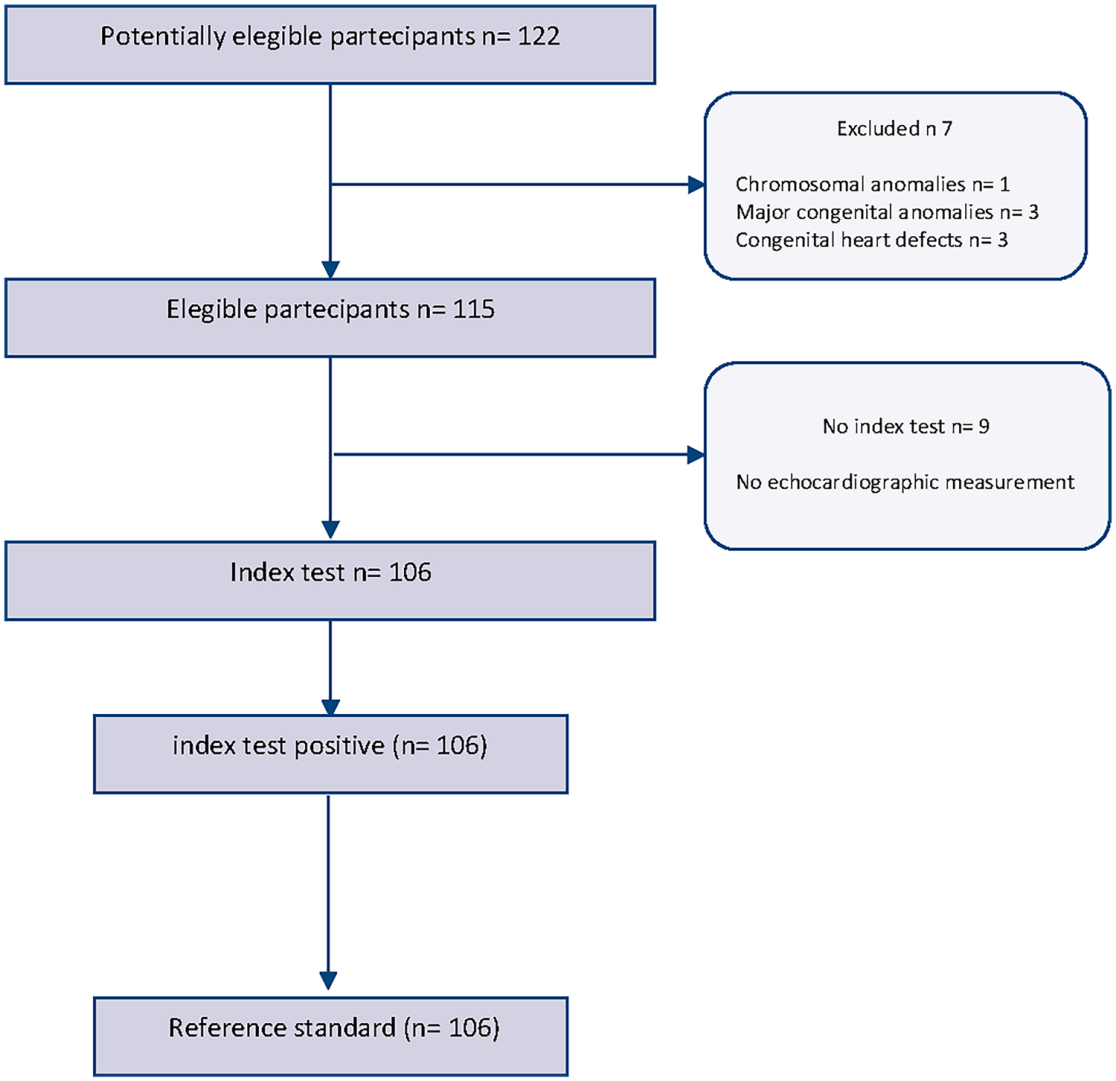
STARD diagram to report flow of participants through the study

Between January 2019 and September 2019, 122 infants from 2 centres were screened for eligibility with a total of 106 infants included in the study (Fig. 2).

Demographic characteristics of the study population are listed in Table 1.

**Table 1:** Baseline characteristics of studied infants (106)

Birth weight (grams)	1504 ± 893
Gestational age (weeks)	30.6 ± 4.0
Apgar score 5’	9 (7–10)
Cesarean section, *n* (%)	83 (78.3)
Antenatal steroids, at least 1 dose, *n* (%)	71 (66.9)
Female, *n* (%)	51 (48.1)
pH at 5 h of life	7.34 (− 7.27–7.41)
Mechanically ventilated on 1st day, *n* (%)	13 (12.2)
Use of inotropes (first 48 h), *n* (%)	5 (4.7)

Values are expressed as mean ± standard deviation, median and interquartile range, number, and percentage

Gaussian distribution was tested by Shapiro–Wilk test (SCVF: *p* < 0.153; UBAF: *p* < 0.0001). In addition, a good correlation was found between SCVF and UBAF (*r* = 0.745; *P* < 0.0001).

**Fig. 3:**
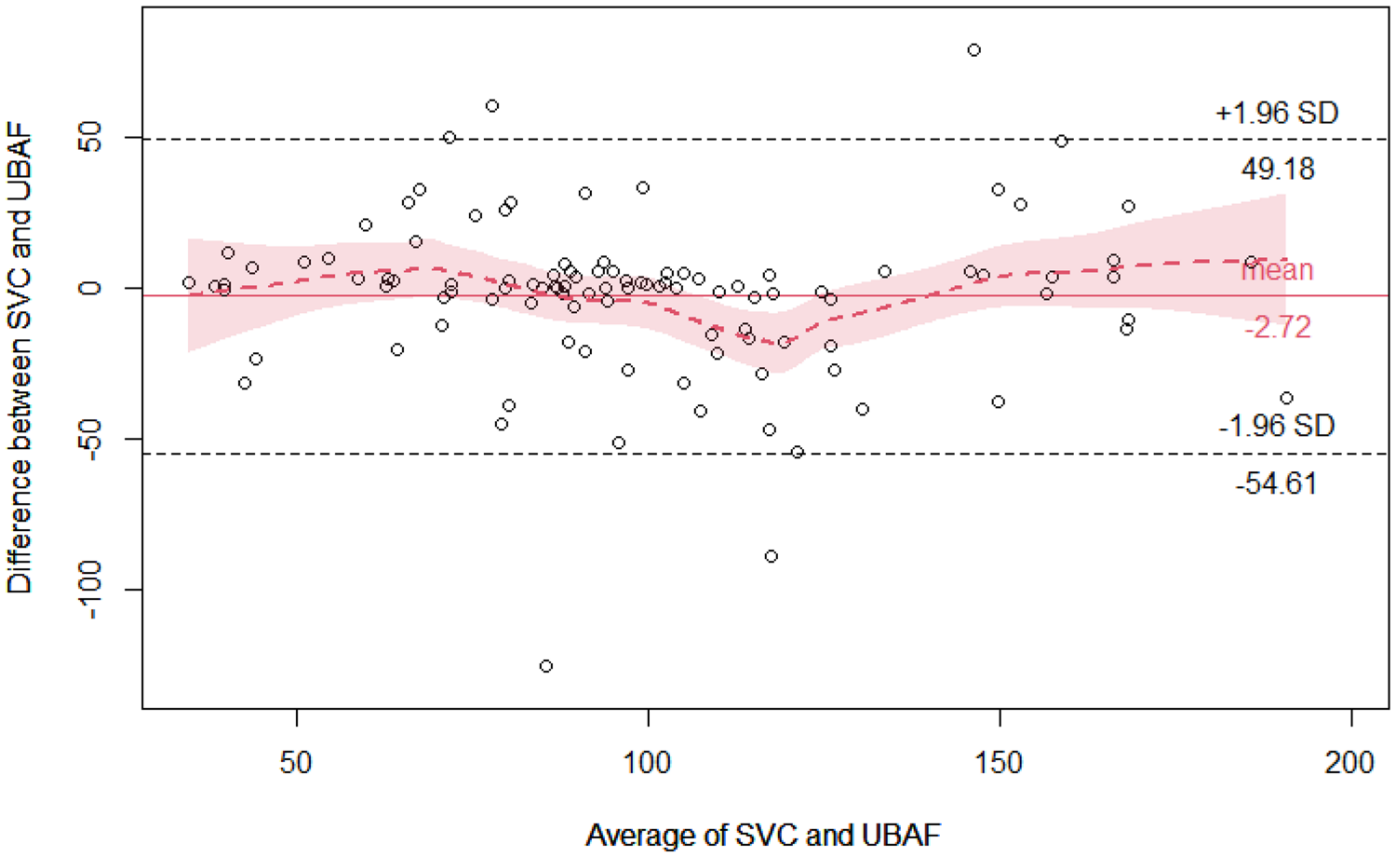
Bla*n*d–Altman analysis: graph with Loess regression. Bias: − 2.717, 95% C.I. [− 2.323; 7.757]; LowLoA − 54.611, 95% C.I. [− 63.352, − 45.869]; UpperLoA 49.177, 95% C.I. [40.43544; 57.91871]

Repeated-measurement Bland–Altman graph analysis showed a good agreement between UBAF and SVCF. The concordance correlation coefficient (CCC) is 0.7434, 95% C.I. [0.656, 0.8111] (Fig. 3). If the analysis excludes 20 examinations defined a priori as of poor quality because of the difficulty of measuring SVCF, an even greater agreement between UBAF and SVCF is observed (CCC 0.8643, 95% C.I. [0.8054, 0.9062]) supporting the hypothesis that some of the variability between the two measures is due to the difficulty of measuring flow in SVC (Table 2).

**Table 2:** Relationship between UBAF and SVCF adjusting for confounding factor (coefficients estimated, standard error (SE), and *p* values)

Variables	Coefficients (SE)	*p*
SVCF	0.866 ± 0.037	< 0.0001
Birthweight	− 10.152 ± 2.540	< 0.0001
Gestational age	1.212 ± 0.506	< 0.0001
PDA	− 10.621 ± 5.093	< 001
UBAF/SVCf	69.364 ± 4.613	< 0.0001

As for the relationship between the two measurement methods, the multiple regression model shows that there is a statistically significant relationship between UBAF and SCVF after adjusting for covariates. The ICC was used to measure the strength of inter-rater agreement. Then, the intra-class correlation coefficient was computed to assess the agreement between two raters in rating the UBAF measures. Finally, there was a good absolute agreement between the two raters ICC = 0.747; p value < 0.0001; 95%CI [0.601; 0.845]. The interrater correlation coefficient could be calculated and the raters’ coefficients of variation (CV) yielded similar values: CV of rater 1 = 38.62%; CV of rater 2 = 38.84%.

## Discussion

The study was designed with the purpose of validating a new and simple method for evaluating for the first time the measurement of UBAF as a substitute SVC flow. The study demonstrated a good agreement between the two methods, considering that perfect equality between UBAF and SVCF was not expected, both because of the presence of non-quantifiable collateral circles of the vascularized districts, and to the inherent limitation of the measurements.

For the purposes of this study, LVO was measured at the level of the ascending aortic arch after assessing a strong correlation between the conventionally measured LVO and LVO at the level of the aortic arch (*r* = 0.897; *P* < 0.0001. CCC 0.7606, 95% C.I. [0.6426, 0.8434]): this choice was made with the aim of evaluating the arterial flow to the upper body through a single echocardiographic view.

The reproducibility of UBAF, both in terms of inter-operator, was found to be better than that of the SVC flow.

A notable issue in assessing the relation between early changes in the systemic blood flow and end-organ injury, particularly to the brain in preterm infants, is how to measure systemic blood flow. Ventricular outputs cannot be used to assess systemic blood flow in preterm infants because they are confounded by shunts through the DA and the atrial septum. The flow returning to the heart through the superior vena cava offers a solution to this problem since it represents flow to the upper body, approximately 80% of which goes to the brain [8]. The SVC flow is used as a systemic perfusion marker in premature infants and has been proposed as a surrogate for cerebral blood flow [6]. Low SVCF in the first postnatal period is related to unfavourable short- and long-term outcomes in infants arousing much interest. Numerous studies have indeed reported an association between a low SVC flow in the first 48 h of life and IVH, adverse neurodevelopmental outcomes and death in preterm infants [9–11].

However, given the risk of errors in the measurement of the SVC diameter, it is not surprising that validation studies on the estimation of SVC flow using echocardiography in newborns have shown poor correlation with cardiac magnetic resonance [12]. The measurement is also burdened by a high intra and inter-observer variability with an error rate of up to 55% [13]. A more recent echocardiographic approach, which contemplates the measure of VTI from the suprasternal window and CSA by tracing the SVC area in the parasternal window at the level of the right pulmonary artery, shows an improvement in the accuracy and repeatability, holding however an error percentage of 36.9 [14–17]. The advantages of measuring an arterial flow are numerous: the arterial vessels, less compressible, do not have variations in diameter with the breath and the cardiac cycle, and the flow rate has minimal changes with respiratory acts; therefore, it does not require to be mediated over several cardiac cycles. Furthermore, clinical conditions that technically impede the measure of SVC flow such as meteorism or marked abdominal distension or in the presence of surgical dressings on the abdomen do not affect the measurement of UBAF. Even frequent anatomical variants such as the persistence of the left SVC, which falsify the measurement of SVC flow, are overcome by the measure of the UBAF. Finally, while SVC flow requires the execution of two different sonographic windows, the evaluation of UBAF can be performed by a single supraclavicular projection.

Limitation of the study was that we compared the new method, UBAF, not with a strong gold standard, such as cardiac MRI or invasive methods such as thermodilution, but with a measure such as the SCVF [18]. The use of different probes and operator expertise could be a limitation despite ICC between operators in a cohort of a population study. The acquisition of flow in the ascending and descending aorta must be done in an appropriate manner, minimising the angle of insonation as much as possible to obtain an adequate VTI. In addition, it may be difficult to place the flow direction upstream of the ductus arteriosus and the choice must be to place it directly downstream of the LSA.

## Conclusion

UBAF showed a strong agreement with SCV flow with a better reproducibility and may be an alternative marker of cerebral perfusion. Further studies are needed to evaluate the normal values of this parameter for gestational age and day of life and its possible applications to clinical practice.

## Data Availability

All data generated or analysed during this study are included in this published article.
